# Ceftazidime-Avibactam Regimens for the Treatment of Bacteremic and Non-Bacteremic Episodes of Carbapenemase-Producing Enterobacterales Infections in Immunosuppressed Patients

**DOI:** 10.3390/pathogens14121300

**Published:** 2025-12-18

**Authors:** Fabián Herrera, Diego Torres, María Leone, Maximiliano Gabriel Castro, Jorge López Camelo, Elena Temporiti, Natalin Grippo, Silvia Relloso, Pablo Bonvehí

**Affiliations:** 1 Infectious Diseases Section, Internal Medicine Department, Centro de Educación Médica e Investigaciones Clínicas, CEMIC, Buenos Aires C1431, Argentina; diegots23@hotmail.com (D.T.); mariavleone@gmail.com (M.L.); maxigabrielcastro@gmail.com (M.G.C.); guchatemporiti@gmail.com (E.T.); pablobonvehi@gmail.com (P.B.); 2 CEMIC-CONICET Research Unit, Centro de Educación Médica e Investigaciones Clínicas, CEMIC, Buenos Aires C1431, Argentina; 3 Microbiology Laboratory, Centro de Educación Médica e Investigaciones Clínicas, CEMIC, Buenos Aires C1431, Argentina; natalingrippo@gmail.com (N.G.); srelloso@cemic.edu.ar (S.R.)

**Keywords:** ceftazidime-avibactam, carbapenemase-producing enterobacterales, immunosuppressed patients

## Abstract

Ceftazidime-avibactam (CA) and CA plus aztreonam (ATM) are the preferred treatment options for KPC and MBL carbapenemase-producing Enterobacterales infections (CPEis). All episodes of monomicrobial CPEis in immunosuppressed patients (IPs) admitted from May 2019 to November 2024, who received definitive antibiotic therapy (AT) with CA or CA + ATM for at least 72 h, were prospectively included. Bacteremic episodes (BEs) and non-bacteremic episodes (NBEs) were compared. Logistic regressions adjusted by propensity score were used to identify variables associated with 30-day overall mortality. In total, 82 CPEis were included (38 NBEs and 44 BEs). BEs more frequently occurred in hematological malignancies (52.3% vs. 15.8%, *p* = 0.0006), while NBEs were more commonly observed in solid organ transplantation (73.7% vs. 34.1%, *p* = 0.001). *K. pneumoniae* was the main isolated microorganism; KPC-CPE was the most common resistance mechanism in both groups, followed by MBL-CPE. The 7-day clinical response, 30-day overall and infection-related mortality between NBEs and BEs were 92.1% vs. 88.6%, *p* = 0.59, 10.5% vs. 27.3%, *p* = 0.09, and 2.6% vs. 13.6%, *p* = 0.11. Septic shock, OR 6.5, 95% CI, 1.58–26.72 (*p* = 0.01), and refractory malignancy, OR 5.6, 95% CI, 1.03–30.14 (*p* = 0.046), were associated with 30-day mortality, whereas BEs were not, OR 1.5, 95% CI, 0.36–6.2 (*p* = 0.56). CPEis in both NBE and BE IPs who received definitive AT with CA or CA + ATM correlated with a high rate of 7-day clinical response and low 30-day infection-related mortality. Underlying malignancy and disease severity were associated with 30-day overall mortality. Regional knowledge of bacterial antibiotic resistance enables the implementation of individualized AT to improve patient survival.

## 1. Introduction

Infections due to carbapenem-resistant enterobacterales (CRE) represent a serious threat worldwide, with *Klebsiella* spp. being the most common isolates. Several countries, such as India, Greece, and Argentina, report the highest incidence of these pathogens [1,2]. The primary resistance mechanism involves carbapenemase-producing enterobacterales (CPE) enzymes, and their prevalence varies across different world regions and countries [3,4,5,6]. Although *K. pneumoniae* carbapenemase (KPC) is still the most common resistance mechanism, Metallo-β-lactamase (MBL) enzymes have emerged globally in recent years and are currently the principal resistant mechanism in CPE in many countries. In this regard, in Argentina, the National Reference Laboratory (NRL) ANLIS/Malbrán reported that among CRE isolates, MBL accounted for 42%, KPC 39.8%, OXA-163 (an OXA-48 variant) 7.4%, and enzyme combinations 8.3% [7]. A mortality > 50% was reported in immunosuppressed patients and those with bacteremia episodes (BEs) and severe clinical presentations [8,9,10,11,12,13,14]. This poor outcome is mainly due to limited therapeutic options and the delay in initiating appropriate antibiotic treatment (AT) [15,16].

In the last decade, new beta-lactams-beta/lactamase inhibitors, such as ceftazidime-avibactam (CA), alone or combined with aztreonam (ATM) for MBL CPE, have been frequently reported to be superior to other antibiotics, like colistin-containing regimens [10,17,18,19,20,21,22,23,24]. Therefore, the IDSA and ESCMID guidelines recommended them as the preferred option for treating CPE infections [25,26].

Over the last few years, several studies have reported on the effectiveness of CA and CA + ATM for the treatment of CPE infections in immunosuppressed patients. They all showed a lower mortality rate than that previously reported in the same population [27,28,29,30]. In addition, a prospective multicenter study revealed that high-risk neutropenic patients with hematologic malignancies (HMs) and hematopoietic cell transplantation (HCT) who had KPC bacteremia and were treated with CA had a lower mortality rate than those treated with other active antibiotic regimens [31].

To the best of our knowledge, and despite real-life data on the effectiveness of CA, no studies in immunosuppressed patients have compared the outcome between patients with BE and NBE infections.

This work aimed to describe and compare clinical, microbiological, and outcome variables in immunosuppressed patients with BE and NBE CPE infections treated with CA regimens. We further analyzed risk factors for 30-day mortality and whether BE itself had an impact on the risk of death.

## 2. Materials and Methods

### 2.1. Setting, Patients, and Study Design

This was a prospective observational study performed at the Center for Medical Education and Clinical Investigation (CEMIC), a university referral hospital specialized in the care of immunosuppressed patients in Argentina.

We evaluated all episodes of CPE infections in adult patients (≥18 years of age) who received definitive AT with CA or CA + ATM from May 2019 to November 2024. The following inclusion criteria were met:

(a) Solid organ transplant (SOT) recipients under immunosuppressive therapy; or
(b) Patients presenting with an HM or solid tumor (ST) treated with recent chemotherapy, anti-lymphocyte therapy, biological agents or high doses of corticosteroids; or
(c) Patients with allogeneic HCT (with graft-versus-host disease at any time or without this condition in the first year post-transplant), or autologous HCT (within 6 months post-transplant); or
(d) Patients presenting with an autoimmune disease under immunosuppressive therapy; and
(e) Those with monomicrobial CPE infections who received definitive AT with CA or CA + ATM for ≥72 h.

The following patients were excluded from the analysis:

(a) Those with recurrent infections;
(b) Those with polymicrobial infections;
(c) Those with positive clinical samples that were not representative of true infection, even if they had received appropriate AT, e.g., asymptomatic bacteriuria, respiratory samples without criteria for pneumonia, superficial skin and soft tissue cultures, and isolation of CPE in blood cultures through the catheter without isolation in peripheral blood cultures;
(d) Those in palliative care.

The study patients were divided into two groups: those with BEs and those with NBEs. They were identified by the Infectious Diseases Section, where all hospitalized patients with CPE treated with CA are evaluated, treated, and followed. The information was provided by the microbiology laboratory and the antimicrobial stewardship program team.

Patients were included in the study at the time of a positive culture and were then prospectively followed on a daily basis during their hospitalization and by phone calls when they were discharged. Data were obtained from direct patient care, electronic medical records, and microbiological laboratory records. Clinical, microbiological, treatment, and outcome variables were evaluated and appropriately defined to avoid inconsistencies, and missing data were not permitted.

The empirical AT was selected either by the physician in charge or by the investigators conducting the study, based on each patient’s clinical and epidemiological features and the presence or not of intestinal colonization by CPE. The investigators carrying out the study chose the definitive therapy based on the CPE type and their antibiotic resistance profile. The personalized treatment duration was consistent with the clinical source and stability. This decision was made in accordance with institutional guidelines and recommendations from IDSA, ESCMID, and ECIL [25,26,32]. Patients were followed for 30 days or until the patient’s death, whichever occurred first.

### 2.2. Definitions

Recent chemotherapy was defined as drug administration for the treatment of HM and ST within a month prior to the infectious episode. High doses of corticosteroids were defined as prednisone (or equivalent) at doses ≥20 mg/day for a period ≥2 weeks prior to the infection, and the use of biological agents and/or anti-lymphocyte therapies, with these drugs administered within six months prior to the infectious episode. Immunosuppressive therapy was defined as the administration of one or more of the following drugs at the time of infection: cyclosporine, mycophenolate mofetil, tacrolimus, sirolimus, and methotrexate.

Neutropenia was defined as an absolute neutrophil count <500 cells/mm^3^. High-risk febrile neutropenia was defined according to the Multinational Association for Supportive Care in Cancer (MASCC) score < 21 and one or more clinical criteria [33,34].

The different types of infections were defined and classified according to the US CDC criteria [35]. Bacteremia was classified as nosocomial, healthcare-associated, or community-acquired according to Friedman et al. [36].

Recent antibiotic use was defined as any antibiotic used 30 days before the infectious episode for more than 48 h. Recent intensive care unit (ICU) admission was defined as an admission within 14 days prior to the episode of infection for at least 72 h. A central venous catheter and a urinary catheter were considered risk factors for CPE when they had been in place for at least 72 h before the infection. Previous hemodialysis, mechanical ventilation, urological instrumentation, and surgery were those procedures performed within 14 days before the infectious episodes.

CPE colonization was defined as “previous” when it occurred within three months prior to hospitalization and “recent” when it was detected during the hospitalization of the infectious episode.

Septic shock was defined as the need for vasopressors to maintain mean arterial pressure ≥ 65 mmHg and serum lactate level >18 mg/dL [37]. Infection severity and mortality probability were defined using Pitt and APACHE-II scores.

Empirical AT was considered appropriate provided that it was started after cultures were taken and one or more antibiotics used were active in vitro against the isolated bacteria, with adequate dosing and dose interval. Empirical AT for bloodstream and urinary tract infections with tigecycline or fosfomycin as monotherapy was deemed inappropriate [25,26]. CA was administered intravenously at a standard dose of 2.5 g every 8 h over a 2 h infusion in patients with normal renal function. In patients with acute kidney injury due to sepsis, doses were adjusted 24 or 48 h after starting therapy [38,39]. Since January 2022, CA has been used in prolonged 3 h infusions [40]. In patients with MBL infections, CA was administered at a dose of 2.5 g every 8 h with ATM at a dose of 2 g every 8 h. Treatment with CA + ATM was considered monotherapy. CA treatment regimens were considered combined therapy when the patient received one or more antibiotics with in vitro activity against the isolated CPE.

Clinical response on day 7 of antibiotic therapy was defined as the absence of fever for at least three days, source control of infection (including catheter or device removal, surgical or percutaneous drainage of infectious collections, and surgical debridement of any tissue), absence of hypotension, and clinical resolution of all signs and symptoms of infection. Mortality was attributable to infection provided that there was microbiological, histological, or clinical evidence of active infection.

### 2.3. Microbiological Studies

Clinical samples were processed according to standardized protocols.

Blood samples were drawn and inoculated in aerobic and anaerobic bottles (BD BACTEC™ Plus Aerobic/F and Plus Anaerobic/F) and monitored in the automatic system BD BACTEC (Becton Dickinson, Sparks, MD, USA) for a minimum incubation period of five days.

Isolates were identified by MALDI-TOF MS (Becton, Dickinson-Bruker Daltonics Biotyper, Billerica, MA, USA). Antibiotic susceptibility testing was performed by disk diffusion, epsilometric tests, and/or the BD Phoenix automated system (Becton Dickinson). Breakpoints and interpretation were according to the CLSI recommendations. Carbapenem resistance was defined as resistance to imipenem, meropenem, or ertapenem, according to NRL ANLIS/Malbrán algorithms [41].

Aztreonam–avibactam synergy was evaluated with the rapid prediffusion technique according to NRL ANLIS/Malbrán recommendations [42]. Carbapenemase production was analyzed with the Blue-carba test and/or disk synergy tests with a carbapenem disk placed close to the boronic acid disk test for KPC, and the EDTA disk for identification of MBL. The presence of genes coding for *bla*KPC and *bla*OXA-48 group was investigated by polymerase chain reaction (PCR) using specific primers. In order to detect colonization with CPE, rectal swabs were routinely collected once a week from the patient’s hospitalization until discharge. The samples were seeded on appropriate chromogenic media (CHROMAgar, Paris, France), and PCR was also performed directly on rectal swabs to detect *bla*KPC and *bla*OXA-48 group.

### 2.4. Statistical Analysis

The study population was characterized by descriptive statistics. For continuous variables, centrality (median) and dispersion (interquartile range [IQR]) measures were used according to the distribution of variables. Categorical variables were analyzed using absolute frequency and percentage. Groups were compared using the Mann–Whitney U-test for continuous variables and Fisher exact test or chi-squared test for categorical variables. For all tests, a 95% level of statistical significance was used.

A multiple logistic regression model was used to identify the risk factors for 30-day mortality. *p* < 0.05 variables in the univariate analysis were included in the multivariate model. Two-tailed *p-*values were reported in all cases.

A comparative analytical study was conducted between patients with BEs and NBEs. A propensity score approach was applied to reduce confounding bias and achieve comparable groups. The propensity score was estimated using a binary logistic regression model, with the dependent variable being the presence of bacteremia (yes/no). Covariates included patient age, APACHE II score, Pitt bacteremia score, and inappropriate empirical antibiotic therapy. These variables were selected based on their biological plausibility and prior evidence of association with infection severity and mortality.

Patients were subsequently stratified according to their propensity scores, using quintiles to ensure adequate comparability between groups. Covariate balance between groups was assessed by comparing standardized mean differences, with an absolute value <0.10 indicating an acceptable level of balance.

A conditional logistic regression model was fitted after stratification to evaluate the association between bacteremia and 30-day mortality (dependent variable), adjusting for potential residual confounders.

Stata software version 18.0 (StataCorp LLC, College Station, TX, USA) was used for the analysis. A two-tailed *p*-value <0.05 was considered statistically significant.

## 3. Results

A total of 177 episodes of CPE infections treated with CA or CA + ATM were evaluated, and 95 were excluded because they failed to meet the eligibility criteria: 83 were not immunosuppressed patients, 11 had polymicrobial infections, and 1 had CA therapy < 48 h. The total study population consisted of 82 patients: 38 (46.4%) had NBEs and 44 (53.6%) had BEs.

Baseline characteristics among patients with NBEs and BEs are outlined in Table 1. Patients with NBEs were older than those with BE. The most frequent underlying diseases in NBE patients were SOT, mainly kidney transplant, compared to HM in the BE group, with acute leukemia being the most common. HCT patients were also more frequent in this group. The underlying malignancies were active in most patients (73%). Regarding the immunosuppressive treatment administered, recent chemotherapy was more frequent in the BE group, compared to other immunosuppressive drugs in the NBE group. High-risk and prolonged febrile neutropenia occurred primarily in patients with BEs.

**Figure 1:**
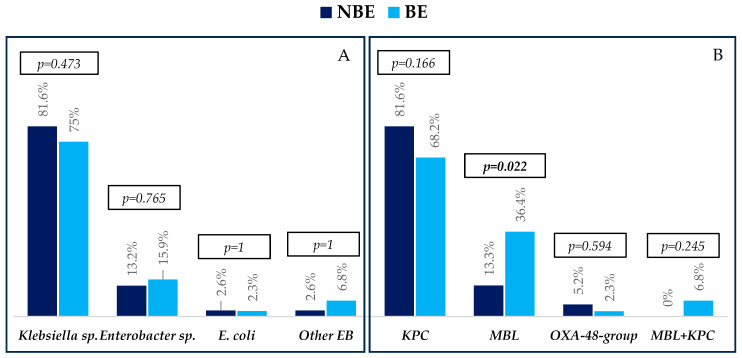
Etiology (**A**) and resistance mechanisms (**B**) of infections in non-bacteremic episodes (NBEs) and bacteremic episodes (BEs). Abbreviations: EB, Enterobacterales; MBL, metallo-beta-lactamase. *p*-values were obtained by chi-squared or Fisher’s exact test.

**Figure 2:**
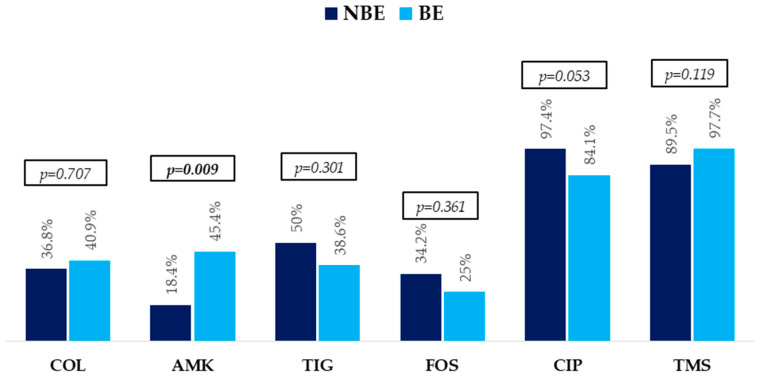
Non-beta-lactam antibiotic resistance profile in non-bacteremic episodes (NBEs) and bacteremic episodes (BEs). *p*-Values were obtained by chi-squared or Fisher’s exact test. Abbreviations: COL, colistin; AMK, amikacin; TIG, tigecycline; FOS, fosfomycin; CIP, ciprofloxacin; TMS, trimethoprim-sulphamethoxazole.

The etiological profiles of bacteremias, resistance mechanisms, and antibiotic resistance profiles are described in Figure 1 and Figure 2.

Regarding the microbiological findings, *Klebsiella* sp. was the bacterium most frequently isolated, followed by *Enterobacter* sp., *Serratia marcescens*, *Escherichia coli*, and *Raoultella ornithinolytica*, with similar distribution between groups. The most common carbapenemase in both groups was KPC, followed by MBL, which was higher in patients with BE. Few patients presented OXA-48-group and KPC+MBL producers.

All isolates of KPC/OXA 48-group and MBL were susceptible to CA and CA + ATM, respectively. They were largely resistant to trimethoprim-sulphamethoxazole and ciprofloxacin. In addition, high resistance to other antibiotics commonly used for treating these infections, such as fosfomycin, tigecycline, colistin, and amikacin, was also observed.

Table 2 describes the epidemiological findings and risk factors for CPE infections.

Most patients had been recently hospitalized and had received AT, which was commonly administered for more than 7 days. Piperacillin-tazobactam and carbapenems were the most frequently prescribed antibiotics, with the latter being used in half of the patients with BE. CPE colonization was present in more than 50% of episodes. The type of carbapenemase involved in decreasing order was KPC, MBL, and OXA-48-group. In all patients, the resistance mechanism of CPE infection was the same as that found in CPE colonization. Most BE patients had a central venous catheter in place, while urologic instrumentation and surgery were most frequently performed in NBE patients.

Clinical characteristics, treatment, and outcomes are described in Table 3.

Almost three quarters of infections were hospital-acquired. Most BE patients had clinical sources, with catheter-related infections only observed in this group. On the other hand, complicated urinary tract infections predominantly occurred in patients with NBEs. Hypotension was common in both groups, and CPE infections presented with fever in more than 90% of patients with BEs. Higher median APACHE II and Pitt scores were reported in patients with BEs.

Fifty-three (64.6%) patients received appropriate empirical AT, with no differences between groups. CA and CA + ATM were prescribed to 34 (41.4%) and 10 (12.1%) of them, respectively. Twenty-four (29.2%) patients received combination therapy, with a higher proportion reported in those with BEs. Other empirical AT herein prescribed included carbapenems, piperacillin-tazobactam, amikacin, and colistin, with the latter two being more frequent in patients with BEs. Combined empirical AT was also more frequently used in this group.

Definitive AT with CA was used in 61 (74.3%) patients, and CA + ATM in 21 (25.6%), and they were both mainly prescribed as monotherapy. The total median duration was 8 days (IQR: 7–10), and no difference was observed between groups.

Regarding outcomes, 19 (23.1%), 16 (19.5%), and 11 (13.4%) patients required ICU admission, developing septic shock and multiorgan failure, respectively. Septic shock was more frequent in patients with BEs; however, no statistically significant difference was observed. The 7-day clinical response, 7-day mortality, 30-day overall, and infection-related mortality were 74 (90.2%), 5 (6.1%), 16 (19.5%), and 7 (8.5%), respectively. Thirty-day mortality was higher in patients with BE, although no statistically significant difference was observed.

Table 4 shows the results of multivariate analyses of risk factors for 30-day mortality.

The independent risk factors for mortality were refractory, underlying malignancy and septic shock development, while bacteremia was not. Similar results were obtained in the survival analysis of patients adjusted by propensity score.

## 4. Discussion

This study evaluated the epidemiological, clinical, and outcomes characteristics of CPE infection episodes with and without bacteremia in immunosuppressed patients who received definitive treatment with CA or CA + ATM. In addition, risk factors for 30-day mortality were identified. The population comprised a cohort of patients who were receiving several immunosuppressive treatments and had high-risk neutropenia in some cases. *Klebsiella* sp. was the main isolated bacterium, and KPC production was the most frequent resistance mechanism involved, followed by MBL production. Complicated urinary tract infection was more frequent in NBE patients, while catheter-related infection was more commonly reported in those with BEs. Intestinal colonization with CPE was the most common risk factor, with the same resistance mechanism as that found in the infection episode. Most of the cohort received monotherapy as definitive AT, for a short duration of time in both groups. Although several patients received inappropriate empirical AT, they had good 7-day clinical response and low 7-day mortality. Thirty-day mortality was higher in patients with BE. Notwithstanding that, in the propensity score-adjusted multivariate model, only septic shock and refractory underlying malignancy were independent risk factors for mortality.

These are the major findings of the present study: the epidemiology of our population shared similarities and differences with that reported in the literature. Regarding SOT recipients, a multicenter study performed in Europe and the USA on patients with CP *K. pneumoniae* found that *bla*KPC was detected in 68.6% of isolates followed by *bla*OXA-48 in 28.6% [14]. Likewise, in a multicenter study from Europe that included bacteremias in neutropenic patients with hematologic malignancies, the carbapenemase most frequently detected was *bla*KPC gene product in 52% of the isolates, followed by *bla*OXA-48 in 28% and *bla*VIM in 7% [29]. On the contrary, in a study carried out in China on patients with hematological diseases and CPE infections, the most commonly expressed genes among the strains were *bla*NDM (66%) and *bla*KPC (29.6%) [28]. Our data showed a different distribution among the CPE infections, with KPC being the most common carbapenemase involved, followed by MBL, and the OXA-48-group being very infrequent. Thus, knowledge of the local epidemiology contributes to a better approach to immunosuppressed patients with these infections.

All isolates were susceptible to CA and CA + ATM but were highly resistant to other antibiotics commonly used for treating these infections. This is in accordance with the data obtained in a prospective multicenter study (RECAPT-AR) conducted in 182 hospitals in Argentina, which collected 821 CRE isolates. Among strains with KPC and MBL production, susceptibility to fosfomycin was 71.5% and 75.1%, tigecycline 64.6% and 64.3%, amikacin 66.8% and 16.6%, and colistin 66.8% and 59.7%, respectively [7]. As largely reported, patients treated with colistin in monotherapy or combined with other antibiotics had a worse outcome than those who received CA and CA + ATM, even when the former were active against the isolated CPE [10,17,19,31]. As previously outlined in our study, this finding is currently more relevant given the high resistance to colistin regimens among EPC isolates. Therefore, colistin regimens are not suitable AT for these patients.

A high prevalence of recent colonization with CPE was observed in our patients. Similar results have been largely reported on this issue. In patients with HM and HCT who develop infections, CPE colonization ranges between 39.2% and 55.6% [11,43]. Moreover, in our study, all CPE infections had the same resistant mechanism as that observed in CPE colonization. This supports the prescription of empirical AT with CA or CA + ATM to immunosuppressed patients colonized by CPE who develop an infection episode.

According to the aforementioned, several measures should be implemented to avoid or lower the risks of CPE colonization, such as infection control measures and antimicrobial stewardships programs, which have proved effective in many studies [44,45,46,47,48]. On the other hand, antibiotic decolonization therapy failed to demonstrate clinical impact on infection rates and mortality and is not currently recommended [49]. Finally, fecal microbiota transplantation has emerged as a promising tool to eradicate CRE colonization [50]. Notwithstanding that, more studies are needed to clarify its role in immunosuppressed patients.

Seventy-three (89.1%) patients in our cohort received definite CA regimens as monotherapy. This approach is consistent with the IDSA and ESCMID guidelines, which recommend against combination therapy for infections caused by CPE, which are susceptible to new beta-lactam–beta-lactamase inhibitors [25,26]. These treatment data are crucial to lower costs and reduce potential drug adverse effects.

Our patients received short AT (median duration 8 days). Some studies carried out in immunocompetent and immunosuppressed patients with multidrug-resistant microorganisms, including CPE, have addressed this issue [10,40,51,52,53,54,55]. This supports the fact that a short course of appropriate AT may be a safe strategy for patients with CPE infections. Therefore, personalized treatment selection has emerged as a promising tool.

A higher rate of patients received inappropriate empirical AT. However, the 7-day clinical response was high, while 7- and 30-day infection-related mortality was not, despite the fact that patients were immunosuppressed and a larger proportion of them presented with severe disease. In our opinion, the fact that the investigators themselves conducted the study, treated all patients, and made—where necessary—early adjustments to antibiotic therapy could have contributed to this scenario.

The 30-day mortality observed in our BE patients appears to be higher than that in NBE subjects. Nevertheless, neither the multivariate analysis nor the propensity score analysis identified bacteremia itself as a risk factor for mortality. On the other hand, only septic shock and refractory malignancy significantly increased the risk. Several studies performed on patients (either immunocompromised or not) with CRE infections showed that septic shock and high Pitt and APACHE II scores were the most important variables associated with disease severity that correlated with 30-day mortality [27,28,29,31]. In view of these findings, we could state that many factors influence mortality and any immunosuppressed patient with CPE infections (with or without BE) is therefore at risk of death.

Our study has some limitations that should be considered. First, our cohort comprised patients with different risks of specific CPE infections, clinical sources, and disease severity. Nevertheless, all of them were immunosuppressed, and therefore at risk of death. Second, although we failed to detect the type of MBL enzyme of CPE isolates, the RECAPT-AR study found that the *bla*NDM gene was present in 98.5% of the strains producing MBL-type enzymes. Third, we did not compare the outcome of patients with KPC producer and MBL producer infections. This could be a bias since the treatment of the latter could be more difficult than that of KPC producers, with a worse outcome. Notwithstanding that, a study reported opposite findings [56]. Fourth, the CPE profile detected in our patients may differ from those in other countries. Therefore, our results could not be extrapolated to other regions. Fifth, although a propensity score analysis was made to reduce the bias in 30-day mortality results, the sample size was small. A larger sample could provide a more accurate outcome between patients with NBEs and BEs.

The strengths of our study rely on its prospective design. It was conducted in a university hospital specialized in the treatment of immunosuppressed patients. All patients with CPE infections who received CA or CA + ATM were evaluated, treated, and followed up by the investigators conducting the study. Therefore, the results herein obtained can accurately represent real-life data on this issue.

## 5. Conclusions

Our study evaluated differences between BE and NBE CPE infections in immunosuppressed patients and their impact on outcomes. They were mostly caused by KPC and MBL CPE. A large number of patients were colonized by CPE presenting the same resistance mechanism as that found in the CPE infection episode. High resistance was observed to the AT most frequently prescribed for these infections. Risk factors for 30-day mortality were associated with underlying malignancy and infection severity rather than with BE itself. Therefore, early and appropriate AT with CA or CA + ATM is crucial in immunosuppressed patients at risk for CPE infections, with or without BE, especially in those with severe clinical presentation. In addition, an individualized approach based on local epidemiology is required to improve patient survival.

## Figures and Tables

**Table 1:** Baseline characteristics.

Variable	NBEs n = 38 n (%)	BEs n = 44 n (%)	Total n = 82 n (%)	*p*-Value
Age, median (IQR)	61 (50–70)	52 (47–62)	56 (47–65)	**0.039**
Male sex	20 (52.6)	28 (63.6)	48 (58.5)	0.313
Charlson comorbidity index, median (IQR)	4 (2–5)	3 (2–5)	3 (2–5)	0.584
Solid organ transplant	28 (73.7)	23 (52.3)	43 (52.4)	**0.0001**
Kidney transplant	25 (65.8)	15 (34.1)	40 (48.8)	**0.004**
Pancreas–kidney transplant	3 (7.9)	0 (0)	3 (3.66)	N/A
Hematological diseases	6 (15.8)	23 (52.3)	29 (35.4)	**0.0006**
Acute myelogenous leukemia	3 (7.9)	12 (27.3)	15 (18.3)	**0.042**
Acute lymphoblastic leukemia	1 (2.6)	4 (9.1)	5 (6.10)	0.366
Multiple myeloma	1 (2.6)	6 (13.6)	7 (8.54)	0.115
Myelodysplastic syndrome	1 (2.6)	1 (2.3)	2 (2.44)	1
HCT	2 (5.2)	9 (20.5)	11 (13.4)	0.055
Autologous	0 (0)	3 (6.8)	3 (3.66)	N/A
Allogeneic	2 (5.2)	6 (13.6)	8 (9.76)	0.275
Solid tumor	4 (10.5)	4 (9.1)	8 (9.76)	1
Autoimmune diseases	2 (5.2)	4 (9.1)	6 (7.32)	0.681
Disease status (in hematologic and solid neoplasms)				
Recent diagnosis	3 (30)	5 (18.5)	8 (9.76)	0.672
Partial remission	1 (10)	1 (3.7)	2 (2.44)	0.473
Refractory	2 (20)	8 (29.6)	10 (12.2)	0.694
Relapsed	1 (10)	6 (22.2)	7 (8.54)	0.647
Complete remission	2 (20)	7 (25.9)	9 (11.0)	1
Immunosuppressive treatment				
Recent chemotherapy	5 (13.2)	22 (50)	27 (32.9)	**<0.0001**
Corticosteroid use	15 (39.5)	11 (25)	26 (31.7)	0.16
Anti-lymphocyte and biological therapies	21 (55.3)	23 (52.3)	44 (53.6)	0.787
Other immunosuppressors	28 (73.7)	17 (38.6)	45 (54.9)	**0.001**
Neutropenia	2 (5.2)	20 (45.4)	22 (26.8)	**<0.0001**
High-risk febrile neutropenia	2 (5.2)	19 (43.2)	21 (25.6)	**0.0001**
Duration of neutropenia, median (IQR)	11 (7–14)	16 (8–24)	15.5 (7.25–22.25)	0.485

Other suppressors: ciclosporin, mycophenolate mofetil or sodium, tacrolimus, sirolimus, and methotrexate. Abbreviation: NBE, non-bacteremic episode; BE, bacteremic episode; IQR, interquartile range; HCT, hematopoietic cell transplant; N/A, not applicable. *p*-Values obtained by chi-squared or Fisher’s exact test for categorical variables, and Mann–Whitney U-test for continuous variables. Bold: statistically significant.

**Table 2:** Epidemiological findings and risk factors for CRE infections.

Variable	NBEs n = 38 n (%)	BEs n = 44 n (%)	Total n = 82 n (%)	*p*-Value
Recent hospitalization	24 (63.2)	27 (61.4)	51 (62.2)	0.867
Previous antibiotic use	32 (84.2)	41 (93.2)	73 (89.0)	0.195
Piperacillin-tazobactam	16 (42.1)	25 (56.8)	41 (50.0)	0.184
Carbapenems	9 (23.7)	22 (50)	31 (37.8)	**0.014**
Ceftazidime-avibactam	8 (21)	8 (18.2)	16 (19.5)	0.947
Previous colonization				
KPC-CRE	14 (36.8)	11 (25)	25 (30.5)	0.245
MBL-CRE	3 (7.9)	6 (13.6)	9 (11.0)	0.494
OXA-48-group—CRE	2 (5.2)	1 (2.3)	3 (3.66)	0.594
Recent colonization				
KPC-CRE	14 (36.8)	23 (52.3)	37 (45.1)	0.161
MBL-CRE	3 (7.9)	9 (20.5)	12 (14.6)	0.129
OXA-48-group—CRE	2 (5.2)	1 (2.3)	2 (2.44)	0.594
Previous ICU admission	8 (21)	17 (38.6)	25 (30.5)	0.085
Previous mechanical ventilation	5 (13.2)	12 (27.3)	17 (20.7)	0.172
Previous hemodialysis	12 (31.6)	8 (18.2)	20 (24.4)	0.159
Previous central venous catheter use	23 (60.5)	42 (95.5)	65 (79.3)	**<0.0001**
Previous urinary catheter use	23 (60.5)	21 (47.7)	44 (53.7)	0.246
Previous urological instrumentation	17 (44.7)	8 (18.2)	25 (30.5)	**0.0009**
Previous surgery	20 (52.6)	13 (29.6)	33 (40.2)	**0.034**

Abbreviation: NBE, non-bacteremic episode; BE, bacteremic episode; CRE, carbapenem-resistant Enterobacterales; MBL, metallo-beta-lactamase; ICU, intensive care unit. *p*-Values obtained by chi-squared or Fisher’s exact test for categorical variables. Bold: statistically significant.

**Table 3:** Clinical characteristics, treatment, and outcomes.

Variable	NBEs n = 38 n (%)	BEs n = 44 n (%)	Total n = 82 n (%)	*p*-Value
Hospital-acquired infection	21 (55.3)	33 (88.6)	60 (73.2)	**0.001**
Healthcare-associated infection	17 (44.7)	5 (11.4)	22 (26.8)	**0.001**
Clinical source				
Primary bacteremia	0 (0)	7 (15.9)	7 (8.54)	N/A
Urinary tract infection	30 (78.9)	13 (29.6)	43 (52.4)	**<0.0001**
Catheter-related infection	0 (0)	7 (15.9)	7 (8.54)	N/A
Abdominal infection	1 (2.6)	10 (22.7)	11 (13.4)	**0.009**
Perianal infection	1 (2.6)	3 (6.8)	4 (4.88)	0.62
Low respiratory tract infection	2 (5.2)	1 (2.3)	3 (3.66)	0.594
Skin and soft tissue infection	3 (7.9)	1 (2.3)	4 (4.88)	0.332
Severe mucositis	0 (0)	4 (9.1)	4 (4.88)	N/A
Fever	24 (63.2)	41 (93.2)	65 (79.3)	**0.001**
Hypotension	8 (21)	16 (36.7)	24 (29.3)	0.129
APACHE II Score—Median (IQR)	14 (11–20)	19 (13–23)	17 (12–21.3)	**0.013**
PITT Score—Median (IQR)	0 (0–2)	1 (0–2)	1 (0–2)	N/A
Empirical AT				
CA	17 (44.7)	17 (38.6)	34 (41.5)	0.576
CA + ATM	2 (5.2)	8 (18.2)	10 (12.2)	0.097
Piperacillin-tazobactam	5 (13.2)	6 (13.6)	11 (13.4)	1
Carbapenem	10 (23.3)	16 (33.4)	26 (31.7)	0.329
Amikacin	2 (5.2)	10 (23.7)	12 (14.6)	**0.031**
Colistin	0 (0)	8 (18.2)	8 (19.4)	N/A
Combined EAT	6 (15.8)	18 (40.9)	24 (29.3)	**0.013**
Appropriated EAT	23 (60.5)	30 (68.2)	53 (64.6)	0.47
Targeted AT				
CA	33 (86.8)	28 (63.6)	61 (74.4)	**0.016**
CA + ATM	5 (13.2)	16 (36.4)	21 (25.6)	**0.022**
Monotherapy	35 (92.1)	38 (86.4)	73 (89)	0.482
Treatment duration (days) (median, IQR)	7 (8–9)	8 (7–11)	8 (7–10)	0.365
Hypotension	8 (21)	16 (36.7)	24 (29.3)	0.129
ICU admission	5 (13.2)	14 (31.8)	19 (23.2)	0.066
Septic shock	4 (10.5)	12 (27.3)	16 (19.5)	0.092
Multiorgan failure	3 (7.9)	8 (18.2)	11 (13.4)	0.208
7-day clinical	35 (92.1)	39 (88.6)	74 (90.2)	0.598
7-day mortality	2 (5.2)	3 (6.8)	5 (6.10)	1
30-day mortality	4 (10.5)	12 (27.3)	16 (19.5)	0.092
Infection-related mortality	1 (2.6)	6 (13.6)	7 (8.54)	0.116

Abbreviations: NBE, non-bacteremic episode; BE, bacteremic episode; IQR, interquartile range; AT, antibiotic treatment; CA, ceftazidime-avibactam; ATM, aztreonam; ICU, intensive care unit; N/A, not applicable. *p*-Values obtained by chi-squared or Fisher’s exact test for categorical variables and Mann–Whitney U-test for continuous variables. Bold: statistically significant.

**Table 4:** Risk factors for 30-day mortality.

Variable	Multivariate Logistic Regression			Multivariate Logistic Regression Adjusted for Propensity Score		
	OR	95% CI	*p*	OR	95% CI	*p*
Bacteremia	1.8	0.44–7.04	0.427	1.5	0.36–6.29	0.567
Septic shock	10.4	2.66–40.8	0.001	6.5	1.58–26.72	0.01
Refractory malignancy	5.5	1.06–28.62	0.043	5.6	1.03–30.14	0.046

## Data Availability

The original contributions presented in this study are included in the article. Further inquiries can be directed to the corresponding author.

## References

[1] Lee Y.L., Ko W.C., Hsueh P.R. (2022). Geographic patterns of global isolates of carbapenem-resistant *Klebsiella pneumoniae* and the activity of ceftazidime/avibactam, meropenem/vaborbactam, and comparators against these isolates: Results from the Antimicrobial Testing Leadership and Surveillance (ATLAS) program, 2020. Int. J. Antimicrob. Agents.

[2] Lin X.C., Li C.L., Zhang S.Y., Yang X.F., Jiang M. (2023). The Global and Regional Prevalence of Hospital-Acquired Carbapenem-Resistant *Klebsiella pneumoniae* Infection: A Systematic Review and Meta-analysis. Open Forum Infect. Dis..

[3] Luterbach C.L., Pasquale D.K., Henderson H.I., Cober E., Richter S.S., Salata R.A., Kalayjian R.C., Watkins R.R., Hujer A.M., Hujer K.M. (2025). A network analysis of carbapenem-resistant *Klebsiella pneumoniae* among healthcare facilities. Sci. Rep..

[4] Kanj S.S., Kantecki M., Arhin F.F., Gheorghe M. (2025). Epidemiology and outcomes associated with MBL-producing Enterobacterales: A systematic literature review. Int. J. Antimicrob. Agents.

[5] Macesic N., Uhlemann A.C., Peleg A.Y. (2025). Multidrug-resistant Gram-negative bacterial infections. Lancet.

[6] Gales A.C., Stone G., Sahm D.F., Wise M.G., Utt E. (2023). Incidence of ESBLs and carbapenemases among Enterobacterales and carbapenemases in Pseudomonas aeruginosa isolates collected globally: Results from ATLAS 2017–2019. J. Antimicrob. Chemother..

[7] Echegorry M., Marchetti P., Sanchez C., Olivieri L., Faccone D., Martino F., Sarkis Badola T., Ceriana P., Rapoport M., Lucero C. (2024). National Multicenter Study on the Prevalence of Carbapenemase-Producing Enterobacteriaceae in the Post-COVID-19 Era in Argentina: The RECAPT-AR Study. Antibiotics.

[8] Gutiérrez-Gutiérrez B., Salamanca E., de Cueto M., Hsueh P.R., Viale P., Paño-Pardo J.R., Venditti M., Tumbarello M., Daikos G., Pintado V. (2016). A Predictive Model of Mortality in Patients with Bloodstream Infections due to Carbapenemase-Producing Enterobacteriaceae. Mayo Clin. Proc..

[9] Xu L., Sun X., Ma X. (2017). Systematic review and meta-analysis of mortality of patients infected with carbapenem-resistant *Klebsiella pneumoniae*. Ann. Clin. Microbiol. Antimicrob..

[10] Balbuena J.P., Cordova E., Mykietiuk A., Farina J., Gañete M., Scapellato P., Lespada M.I., Nannini E., Contreras R., Cunto E. (2025). Carbapenem-resistant Gram-negative bacilli bacteremia in Argentina (EMBARCAR): Findings from a prospective, multicenter cohort study. Clin. Infect. Dis..

[11] Freire M.P., Pierrotti L.C., Filho H.H., Ibrahim K.Y., Magri A.S., Bonazzi P.R., Hajar L., Diz M.P., Pereira J., Hoff P.M. (2015). Infection with *Klebsiella pneumoniae* carbapenemase (KPC)-producing *Klebsiella pneumoniae* in cancer patients. Eur. J. Clin. Microbiol. Infect. Dis..

[12] Trecarichi E.M., Pagano L., Martino B., Candoni A., Di Blasi R., Nadali G., Fianchi L., Delia M., Sica S., Perriello V. (2016). Haematologic Malignancies Associated Bloodstream Infections Surveillance (HEMABIS) registry—Sorveglianza Epidemiologica Infezioni Funginein Emopatie Maligne (SEIFEM) group, Italy. Bloodstream infections caused by *Klebsiella pneumoniae* in onco-hematological patients: Clinical impact of carbapenem resistance in a multicentre prospective survey. Am. J. Hematol..

[13] Tofas P., Skiada A., Angelopoulou M., Sipsas N., Pavlopoulou I., Tsaousi S., Pagoni M., Kotsopoulou M., Perlorentzou S., Antoniadou A. (2016). Carbapenemase-producing *Klebsiella pneumoniae* bloodstream infections in neutropenic patients with haematological malignancies or aplastic anaemia: Analysis of 50 cases. Int. J. Antimicrob. Agents.

[14] Pérez-Nadales E., Fernández-Ruiz M., Gutiérrez-Gutiérrez B., Pascual Á., Rodríguez-Baño J., Martínez-Martínez L., Aguado J.M., Torre-Cisneros J. (2022). Extended-spectrum β-lactamase-producing and carbapenem-resistant Enterobacterales bloodstream infection after solid organ transplantation: Recent trends in epidemiology and therapeutic approaches. Transpl. Infect. Dis..

[15] Falcone M., Bassetti M., Tiseo G., Giordano C., Nencini E., Russo A., Graziano E., Tagliaferri E., Leonildi A., Barnini S. (2020). Time to appropriate antibiotic therapy is a predictor of outcome in patients with bloodstream infection caused by KPC-producing *Klebsiella pneumoniae*. Crit. Care.

[16] Micozzi A., Gentile G., Minotti C., Cartoni C., Capria S., Ballarò D., Santilli S., Pacetti E., Grammatico S., Bucaneve G. (2017). Carbapenem-resistant *Klebsiella pneumoniae* in high-risk haematological patients: Factors favouring spread, risk factors and outcome of carbapenem-resistant *Klebsiella pneumoniae* bacteremias. BMC Infect. Dis..

[17] van Duin D., Lok J.J., Earley M., Cober E., Richter S.S., Perez F., Salata R.A., Kalayjian R.C., Watkins R.R., Doi Y. (2018). Colistin Versus Ceftazidime-Avibactam in the Treatment of Infections Due to Carbapenem-Resistant Enterobacteriaceae. Clin. Infect. Dis..

[18] Castón J.J., Cano A., Pérez-Camacho I., Aguado J.M., Carratalá J., Ramasco F., Soriano A., Pintado V., Castelo-Corral L., Sousa A. (2022). Impact of ceftazidime/avibactam versus best available therapy on mortality from infections caused by carbapenemase-producing Enterobacterales (CAVICOR study). J. Antimicrob. Chemother..

[19] Falcone M., Giordano C., Leonildi A., Galfo V., Lepore A., Suardi L.R., Riccardi N., Barnini S., Tiseo G. (2024). Clinical Features and Outcomes of Infections Caused by Metallo-β-Lactamase-Producing Enterobacterales: A 3-Year Prospective Study from an Endemic Area. Clin. Infect. Dis..

[20] Karaiskos I., Daikos G.L., Gkoufa A., Adamis G., Stefos A., Symbardi S., Chrysos G., Filiou E., Basoulis D., Mouloudi E. (2021). Ceftazidime/avibactam in the era of carbapenemase-producing *Klebsiella pneumoniae*: Experience from a national registry study. J. Antimicrob. Chemother..

[21] Zhuang H.H., Qu Q., Long W.M., Hu Q., Wu X.L., Chen Y., Wan Q., Xu T.T., Luo Y., Yuan H.Y. (2025). Ceftazidime/avibactam versus polymyxin B in carbapenem-resistant *Klebsiella pneumoniae* infections: A propensity score-matched multicenter real-world study. Infection.

[22] Chen Y., Huang H.B., Peng J.M., Weng L., Du B. (2022). Efficacy and Safety of Ceftazidime-Avibactam for the Treatment of Carbapenem-Resistant *Enterobacterales* Bloodstream Infection: A Systematic Review and Meta-Analysis. Microbiol. Spectr..

[23] Chen J., Hu Q., Zhou P., Deng S. (2024). Ceftazidime-avibactam versus polymyxins in treating patients with carbapenem-resistant Enterobacteriaceae infections: A systematic review and meta-analysis. Infection.

[24] Gupta N., Boodman C., Prayag P., Manesh A., Kumar T.P. (2024). Ceftazidime-avibactam and aztreonam combination for Carbapenem-resistant Enterobacterales bloodstream infections with presumed *Metallo-β-lactamase* production: A systematic review and meta-analysis. Expert Rev. Anti-Infect. Ther..

[25] Tamma P.D., Heil E.L., Justo J.A., Mathers A.J., Satlin M.J., Bonomo R.A. (2024). Infectious Diseases Society of America 2024 Guidance on the Treatment of Antimicrobial-Resistant Gram-Negative Infections. Clin. Infect. Dis..

[26] Paul M., Carrara E., Retamar P., Tängdén T., Bitterman R., Bonomo R.A., de Waele J., Daikos G.L., Akova M., Harbarth S. (2022). European Society of Clinical Microbiology and Infectious Diseases (ESCMID) guidelines for the treatment of infections caused by multidrug-resistant Gram-negative bacilli (endorsed by European society of intensive care medicine). Clin. Microbiol. Infect..

[27] Tumbarello M., Giuliano G., Criscuolo M., Del Principe M.I., Papayannidis C., Fracchiolla N.S., Dargenio M., Cefalo M., Nadali G., Candoni A. (2025). Clinical impact of ceftazidime/avibactam on the treatment of suspected or proven infections in a large cohort of patients with haematological malignancies: A multicentre observational real-world study. J. Antimicrob. Chemother..

[28] Zhang L., Zhen S., Shen Y., Zhang T., Wang J., Li J., Lin Q., Xiao Z., Zheng Y., Jiang E. (2023). Bloodstream infections due to Carbapenem-Resistant Enterobacteriaceae in hematological patients: Assessment of risk factors for mortality and treatment options. Ann. Clin. Microbiol. Antimicrob..

[29] Sastre-Escolà E., Ntziora F., Chiurlo M., Martín-Dávila P., Mikulska M., Albasanz-Puig A., Machado M., Gutiérrez-Villanueva A., Márquez-Gómez I., Gasch-Blasi O. (2025). Ceftazidime/avibactam for the treatment of bloodstream infection due to carbapenemase-producing Enterobacterales in onco-haematologic neutropenic patients (the TARZAN study). J. Antimicrob. Chemother..

[30] Hu J., Zha L., Yu Y.W., Su Q., Fang X.L., Ji J.R., Shen P., Chen Y.B., Zheng X., Xiao Y.H. (2024). Efficacy of ceftazidime-avibactam in the treatment of carbapenem-resistant *Klebsiella pneumoniae* infections: Focus on solid organ transplantation recipients. Int. J. Antimicrob. Agents.

[31] Herrera F., Torres D., Laborde A., Jordán R., Mañez N., Berruezo L., Lambert S., Suchowiercha N., Costantini P., Nenna A. (2024). Ceftazidime-Avibactam Improves Outcomes in High-Risk Neutropenic Patients with *Klebsiella pneumoniae* Carbapenemase-Producing Enterobacterales Bacteremia. Microorganisms.

[32] Averbuch D., Cordonnier C., Livermore D.M., Mikulska M., Orasch C., Viscoli C., Gyssens I.C., Kern W.V., Klyasova G., Marchetti O. (2013). Targeted therapy against multi-resistant bacteria in leukemic and hematopoietic stem cell transplant recipients: Guidelines of the 4th European Conference on Infections in Leukemia (ECIL-4, 2011). Haematologica.

[33] Freifeld A.G., Bow E.J., Sepkowitz K.A., Boeckh M.J., Ito J.I., Mullen C.A., Raad I.I., Rolston K.V., Young J.A., Wingard J.R. (2011). Clinical practice guideline for the use of antimicrobial agents in neutropenic patients with cancer: 2010 update by the Infectious Diseases Society of America. Clin. Infect. Dis..

[34] Averbuch D., Orasch C., Cordonnier C., Livermore D.M., Mikulska M., Viscoli C., Gyssens I.C., Kern W.V., Klyasova G., Marchetti O. (2013). European guidelines for empirical antibacterial therapy for febrile neutropenic patients in the era of growing resistance: Summary of the 2011 4th European Conference on Infections in Leukemia. Haematologica.

[35] Horan T.C., Andrus M., Dudeck M.A. (2008). CDC/NHSN surveillance definition of health care-associated infection and criteria for specific types of infections in the acute care setting. Am. J. Infect. Control.

[36] Friedman N.D., Kaye K.S., Stout J.E., McGarry S.A., Trivette S.L., Briggs J.P., Lamm W., Clark C., MacFarquhar J., Walton A.L. (2002). Health care-associated bloodstream infections in adults: A reason to change the accepted definition of community-acquired infections. Ann. Intern. Med..

[37] Singer M., Deutschman C.S., Seymour C.W., Shankar-Hari M., Annane D., Bauer M., Bellomo R., Bernard G.R., Chiche J.D., Coopersmith C.M. (2016). The Third International Consensus Definitions for Sepsis and Septic Shock (Sepsis-3). JAMA.

[38] Bidell M.R., Lodise T.P. (2018). Suboptimal Clinical Response Rates with Newer Antibiotics Among Patients with Moderate Renal Impairment: Review of the Literature and Potential Pharmacokinetic and Pharmacodynamic Considerations for Observed Findings. Pharmacotherapy.

[39] Crass R.L., Rodvold K.A., Mueller B.A., Pai M.P. (2019). Renal Dosing of Antibiotics: Are We Jumping the Gun?. Clin. Infect. Dis..

[40] Tumbarello M., Raffaelli F., Giannella M., Mantengoli E., Mularoni A., Venditti M., De Rosa F.G., Sarmati L., Bassetti M., Brindicci G. (2021). Ceftazidime-Avibactam Use for *Klebsiella pneumoniae* Carbapenemase-Producing *K. pneumoniae* Infections: A Retrospective Observational Multicenter Study. Clin. Infect. Dis..

[41] http://antimicrobianos.com.ar/ATB/wp-content/uploads/2021/11/ALGORITMO-ETB-2021.pdf.

[42] https://antimicrobianos.com.ar/ATB/wp-content/uploads/2021/03/Predifusion-rapida-ATM-AVI.pdf.

[43] Girmenia C., Rossolini G.M., Piciocchi A., Bertaina A., Pisapia G., Pastore D., Sica S., Severino A., Cudillo L., Ciceri F. (2015). Infections by carbapenem-resistant *Klebsiella pneumoniae* in SCT recipients: A nationwide retrospective survey from Italy. Bone Marrow Transplant..

[44] Viale P., Tumietto F., Giannella M., Bartoletti M., Tedeschi S., Ambretti S., Cristini F., Gibertoni C., Venturi S., Cavalli M. (2015). Impact of a hospital-wide multifaceted programme for reducing carbapenem-resistant Enterobacteriaceae infections in a large teaching hospital in northern Italy. Clin. Microbiol. Infect..

[45] Enfield K.B., Huq N.N., Gosseling M.F., Low D.J., Hazen K.C., Toney D.M., Slitt G., Zapata H.J., Cox H.L., Lewis J.D. (2014). Control of simultaneous outbreaks of carbapenemase-producing enterobacteriaceae and extensively drug-resistant *Acinetobacter baumannii* infection in an intensive care unit using interventions promoted in the Centers for Disease Control and Prevention 2012 carbapenemase-resistant Enterobacteriaceae Toolkit. Infect. Control Hosp. Epidemiol..

[46] Schwaber M.J., Lev B., Israeli A., Solter E., Smollan G., Rubinovitch B., Shalit I., Carmeli Y. (2011). Israel Carbapenem-Resistant Enterobacteriaceae Working Group. Containment of a country-wide outbreak of carbapenem-resistant Klebsiella pneumoniae in Israeli hospitals via a nationally implemented intervention. Clin. Infect. Dis..

[47] López-Viñau T., Peñalva G., García-Martínez L., Castón J.J., Muñoz-Rosa M., Cano Á., Recio M., Cisneros J.M., Pérez-Nadales E., Rumbao Aguirre J. (2021). Impact of an Antimicrobial Stewardship Program on the Incidence of Carbapenem Resistant Gram-Negative Bacilli: An Interrupted Time-Series Analysis. Antibiotics.

[48] Seas C., Legua P., Delfin B., Villavicencio K., Palomino A., Montenegro P., Aguilar I., La Rosa Y., Robles M., Young F. (2024). Implementing an Antimicrobial Stewardship Program in an Oncology Center in Lima, Peru: A Model for Low- and Middle-Income Countries. Open Forum Infect. Dis..

[49] Rodríguez Feria D., Diaz Brochero C.R., Muñoz Velandia O., Verhelst López J.M., Garzón Herazo J.R. (2025). Effectiveness and safety of oral antibiotics as a decolonization strategy for carbapenem-resistant Enterobacteriaceae: A systematic review of randomized and non-randomized studies. Infect. Dis. Now.

[50] Macareño-Castro J., Solano-Salazar A., Dong L.T., Mohiuddin M., Espinoza J.L. (2022). Fecal microbiota transplantation for Carbapenem-Resistant Enterobacteriaceae: A systematic review. J. Infect..

[51] You T.Y., Lo C.L., Tsai W.C., Jan H.E., Ko W.C., Lee N.Y. (2024). Efficacy of short- versus prolonged-courses of antimicrobial therapy for carbapenem-resistant *Klebsiella pneumoniae* bloodstream infections: A propensity score-matched cohort study. J. Microbiol. Immunol. Infect..

[52] Soto C.L., Hsu A.J., Lee J.H., Dzintars K., Choudhury R., Jenkins T.C., McCreary E.K., Quartuccio K.S., Stohs E.J., Zimmerman M. (2024). Identifying Effective Durations of Antibiotic Therapy for the Treatment of Carbapenem-resistant Enterobacterales Bloodstream Infections: A Multicenter Observational Study. Clin. Infect. Dis..

[53] Avni-Nachman S., Yahav D., Nesher E., Rozen-Zvi B., Rahamimov R., Mor E., Ben-Zvi H., Milo Y., Atamna A., Green H. (2021). Short versus prolonged antibiotic treatment for complicated urinary tract infection after kidney transplantation. Transpl. Int..

[54] Imlay H., Spellberg B. (2022). Shorter is better: The case for short antibiotic courses for common infections in solid organ transplant recipients. Transpl. Infect. Dis..

[55] Herrera F., Torres D., Laborde A., Jordán R., Tula L., Mañez N., Pereyra M.L., Suchowiercha N., Berruezo L., Gudiol C. (2024). Seven-day antibiotic therapy for Enterobacterales bacteremia in high-risk neutropenic patients: Toward a new paradigm. Eur. J. Clin. Microbiol. Infect. Dis..

[56] Seo H., Kim H.J., Kim M.J., Chong Y.P., Kim S.H., Lee S.O., Choi S.H., Kim Y.S., Woo J.H., Jung J. (2021). Comparison of clinical outcomes of patients infected with KPC- and NDM-producing Enterobacterales: A retrospective cohort study. Clin. Microbiol. Infect..

